# Clinical Outcomes of the Ilizarov Method After an Infected Tibial Non Union

**DOI:** 10.5812/atr.11300

**Published:** 2013-08-01

**Authors:** Mohammad Shahid, Abid Hussain, Phillipa Bridgeman, Deepa Bose

**Affiliations:** 1 Limb Reconstruction Unit, Queen Elizabeth Hospital, Birmingham, UK; 2 Queen Elizabeth Hospital, Birmingham, UK

**Keywords:** Ilizarov, Tibial Non Union, Osteomyelitis

## Abstract

**Background:**

The Ilizarov technique has been used in the UK for the last 20 years in the management of infected non-union of long bones. This method uses fine wires inserted percutaneously which are attached and tensioned to provide a strong frame construct. The majority of tibial and femoral non unions can be treated successfully by internal fixation. However, an infected non-union of the tibia can prove a difficult problem. The Ilizarov method can prove useful for treating these complex injuries.

**Objectives:**

To assess whether a new limb reconstruction centre in the UK has comparable results.

**Patients and Methods:**

Twelve patients (10 M: 2 F; Avg age 43.3 years) who had an infected tibial non-union between March 2009 and August 2010 treated with the Ilizarov technique. Intervention method was Ilizarov technique and main outcome measures include functional and radiological outcomes assessed using the Association for the Study and Application of Methods of Ilizarov (ASAMI) criteria, American Orthopaedic Foot and Ankle Score (AOFAS) and Visual Analogue Pain scores.

**Results:**

All twelve patients united. None required amputation. Mean time to union was 46 weeks (range 24 - 70/median 50). The average follow up time was 62 weeks (39 - 164/ median 59). According to the ASAMI score bone/radiological results ten were classed as excellent with the remainder being good. Functionally six were graded as excellent, four as good and two as poor. The average AOFAS score was 83/100 (70 - 90) and pain visual analogue scale (VAS) was two.

**Conclusions:**

Our results in terms of ASAMI scores are comparable with the published literature. Furthermore, our return to work is better than most European studies (63%). All our patients said they would have the procedure again. We attribute this success partly to the multidisciplinary approach. We recommend early referral to a dedicated unit if there is any evidence of a non-union.

## 1. Background

The Ilizarov technique has been used in the UK for the last 20 years in the management of infected non-union of long bones. This method uses fine wires inserted percutaneously which are attached and tensioned to provide a strong frame construct (1-3). It permits the use of compression, distraction, bone lengthening and deformity correction. Due to the need for specialist surgery and post-operative rehabilitation its use is confined to specialist centres. The majority of tibial and femoral non unions can be treated successfully by internal fixation. However, an infected non-union of the tibia can prove a difficult problem. This can be compounded by bone loss, deformity or failure of previous internal fixation. The choice of such procedure can mean the difference between limb salvage and amputation (4). The treatment of bone infections after intramedullary nailing usually includes a series of different surgical procedures such as removal of metalwork, radical bony debridement, deep tissue sampling, elimination of dead space and insertion of local antibiotic delivery systems. This is followed by the application of the Ilizarov external fixator. Furthermore, local or free soft tissue transfers are employed to cover any soft tissue defect. The Ilizarov method addresses all the above problems simultaneously and offers a good solution for infected non unions. The stability of the construct permits weight bearing and joint mobilisation. Furthermore, bone defects can be filled by a corticotomy and bone transport. The control of infection is achieved by radical debridement of the bone ends (5). Amputation is one of the risks of infected non-union and so the Ilizarov method can minimise this potential outcome (3).

## 2. Objectives

The Birmingham Limb Reconstruction was set up in January 2009. It is a relatively new unit and we wanted to see if our results were on par, if not better than other established units. If they are better then why is this so?

## 3. Patients and Methods

### 3.1. Inclusion Criteria

All patients who had an infected tibial non-union at the senior author’s hospital between March 2009 and August 2010 were included in this study. Initially there were twenty patients but only twelve could be followed up (Table 1). 

**Table 1.** Patient Demographics

Features	Variant
**Age (range), y**	43.3 (28 - 89)
**Gender (M:F)**	10:2
**Site of initial fracture**	Prox 2, Mid 7, Distal 3
**Alcoholic/Substance abuser/Smokers/NSAIDs/Steroids, No.**	1/1/6/0/1
**Cierny and Mader Host Classification **	A: 6, B:6, C:0
**Open Fractures-Gustilo Grading **	G 3B four : G 2 one
**Previous procedures**	IM nail 7 ^a^; plating 4; ex fix 2
**Time from injury to frame, wk, Mean/Median, (range)**	45/23 (2 - 149)
**Time from referral to frame, wk , Mean/Median, (range)**	21/21 (1 - 62)
**Follow up time, wk ,Mean/Median, (range)**	62/59 (39 - 164)
**time to union, wk ,Mean/Median, (range)**	46/50 (24 - 70)
**Post Frame Complications**	Pin site infections x2Donor flap seroma x1Broken wire x1

^a^ One patient had an exchange nailing at the referring hospital

### 3.2. Exclusion Criteria

There were ten men and two women with an average age of 43.3 years (range 28 - 89). Four referrals were internal and the remainders were from other hospitals in the region. In terms of significant comorbidities there was one alcoholic and one who was a long term substance user. Six were smokers and one was on an inhaled steroid for their asthma. Using Cierny and Mader Host classification six were A and the remainder were B (6). In seven patients the fracture was at the midshaft, two proximally and three distal. Previous procedures were seven intramedullary nailings, four platings and two monolateral external fixators. One patient had an exchange nailing. Five were open injuries (3B – four, 2 – one) as per Gustilo and Anderson classification (7).

The average time from initial injury to application of an Ilizarov frame was 45 weeks (range 2 to 149 /median 23. The average time from referral to application of the frame was 21 weeks (range 1 to 62 /median 21). All definitive procedures were done by a single surgeon (DB). All cases were discussed and treated by a multidisciplinary team of orthopaedic and plastic surgeons, radiologist and microbiologist, specialist nurse and physiotherapist. At final follow up MS and AH who were not involved in the surgical treatment assessed the patients functionally and radiologically. Functional and radiological outcomes were assessed using the Association for the Study and Application of Methods of Ilizarov (ASAMI) criteria (8). We also measured the American Orthopaedic Foot and Ankle Score (AOFAS) (9) which incorporates subjective and objective criteria and is graded with a maximum score of 100 of which 50 are allocated to function, 40 to pain and 10 to alignment.

### 3.3. Surgical Technique

The initial metalwork was removed and the tibia was reamed in those patients who had previously had an intramedullary nail. The bone ends were debrided and six (5 for micro + 1 for histology) deep tissue samples were taken using separate instruments for each sample. These samples comprised any soft tissue and bone from the non-union site and were subsequently sent for culture. The frame was then applied with transosseous wires and half pins to preserve the anatomical axis and avoid any additional soft tissue damage. The frames were extended to the foot to minimize equinus deformity where necessary. If soft tissue coverage was required then this was done by the plastic surgeons at the same sitting. Post operatively, the patients were encouraged to weight bear immediately with the aid of crutches. As soon as samples were taken in theatre they were started on Vancomycin and Meropenem and these were continued until sensitivities were back. The antibiotic plan was then formulated with the microbiologists. Patients were then subsequently discharged into the community.

## 4. Results

All twelve patients eventually united. None required amputation. The mean time to union was 46 weeks (range 24 to 70/median 50). The average follow up time was 62 weeks (39 to 164/median 59). The average leg length discrepancy was 0.8 cm (0 to 2). Six had an obvious limp. One had an appreciable ankle equinus and four had soft tissue dystrophy. Two patients developed pin site infections which were successfully treated with oral antibiotics. One had a seroma from the flap donor site which was subsequently aspirated and there was one broken wire which was replaced. In terms of organisms five patients grew Staph. Aureus, two grew Coagulase Negative Staph and one grew Strept Viridans. One grew a Bacillus species, one Serratia marcescens and one grew Propionibacterium with Stenotrophomonas (Pseudomonas) maltophilia. One patient had a negative culture but he was on antibiotics preoperatively and it was noticed intraoperatively that there was a biofilm around his implant. This was subsequently treated as a culture negative infected non union

### 4.1. Scoring

The second questionnaire for ankle status was the AOFAS (American Foot and Ankle Hindfoot Scale) ( 9 ). On this a higher score reflects better pain control and function. The average pain score was 36 / 40. The average function score was 38 / 50 and the mean alignment score was 9 / 10. Overall the average total score was 83 / 100 (range 70 to 90) **(Table 2, Table 3 and Table 4). 

**Table 2.** Bone Results Using the Association for the Study and Application of Methods of Ilizarov Scoring System

Bone Results	Description	Score
**Excellent**	Union, no infection, deformity < 7, limb length discrepancy < 2.5 cm	10
**Good**	Union + any two of the following: absence of infection < 7 deformity and limb length inequality of < 2.5 cm	2
**Fair**	Union + only one of the following: absence of infection, deformity < 7 and limb length inequality < 2.5 cm	0
**Poor**	Non union/refracture/union + infection + deformity > 7 + limb length inequality > 2.5cm	0

**Table 3.** Functional Results Using the Association for the Study and Application of Methods of Ilizarov Scoring System

Functional	Description	Score
**Excellent**	Active, no limp, minimum stiffness (loss of < 15 knee extension/ < 15 dorsiflexion of ankle), no reflex sympathetic dystrophy (RSD), insignificant pain	6
**Good**	Active, with one or two of the following: limp, stiffness, RSD ^a^, significant pain	4
**Fair**	Active, with three or all of the following: limp, stiffness, RSD ^a^, significant pain	0
**Poor**	Inactive (unemployment or inability to perform daily activities because of injury)	2
**Failures**	Amputation	0

^a^ Abbreviation: RSD, reflex sympathetic dystrophy

**Table 4.** AOFAS ^a ^and pain VAS ^a ^scores

	Score, No. (Range)
**Pain**	36 (30 - 40)
**Function**	38 (30 - 40)
**Alignment**	9 (8 - 15)
**Overall AOFAS ** ^**a**^ ** score**	83 (70 - 90)
**Average Pain Visual Analogue Score**	2 (0 - 4)

^a^Abbreviations: AOFAS, american orthopaedic foot and ankle score; VAS, visual analogue scale

All patients said they would have the procedure again. According to the ASAMI score bone/radiological results ten were classed as excellent with the remainder being good. Functionally six were graded as excellent, four as good and two as poor. The ones classed as poor found it difficult to go back to work. In terms of employment seven went back to their original jobs. One lost his job as a plumber after the injury and found it difficult to find further employment. He said he didn’t feel his injury restricted him from work. One was on a retirement pension. One was unemployed before this injury and remained that way. Two were unable to work as they were manual workers and found it difficult to kneel.

## 5. Discussion

The Ilizarov technique has been utilised in the UK for over 15 years. It offers an effective and reliable treatment for some of the most challenging conditions in orthopaedics such as infected tibial non unions. It was initially developed by Gavril Ilizarov in Kurgan, Russia in the 1950s but only rose to prominence after treating Russian high jump god medallist Valery Brummell after an infected tibial non-union. Infected tibial non unions are a complicated problem requiring complex time consuming surgery. We have demonstrated that good function can be achieved in terms of union, pain relief, activities of daily living and employment. However, this is a long and arduous process requiring patient compliance. Previous papers looking at the use of the Ilizarov technique (Table 5) have shown that our results using the ASAMI criteria are on par with all and better than most. Many of the other papers have incorporated non unions without any evidence of infection. These we would expect to have better outcomes as the patient would not require such radical debridement and treatment of osteomyelitis. However, we have shown our results to be superior. The reasons for this could be our meticulous taking of deep tissue samples. We use a different set of instruments for each sample so as to minimise contamination. Furthermore, we take five samples so that decision-making is easier regarding antibiotic treatment if equal numbers of samples are positive. We then tailor our antibiotics to the results. Regarding returning to work, in our series eight of the twelve went back to their original jobs. One lost his job as a plumber and found it difficult to find other work due to the economic climate but he did not feel his injury impeded him in anyway. One patient was a heating engineer and could not go back to his original job as he found kneeling down difficult. One was 86 at the time and subsequently retired. One was unemployed prior to the injury and was not actively looking for work. Our figures show 63% went back to work. 

**Table 5.** A Comparison of the Results Using the Association for the Study and Application of Methods of Ilizarov Criteria

Authors	Subjects Studied	ASAMI ^a^ Bone Scores, % E,G,F,P ^a^	ASAMI Functional Scores, % E,G,F,P ^a^	Return to Work, %
				
**Dendrinos et al.**	Infected tibial non unions	50, 28, 4, 18	26, 41, 15, 18	82
**Maini et al.**	Infected non unions	70, 10, 0, 20	27, 40, 10, 23	77
**Patil et al.**	Infected tibial and femoral non unions	42, 34,10, 14	44, 44, 6, 6	22
**Present study**	Infected tibial non unions	83, 17, 0, 0	50, 33, 0, 17	63

^a^ Abbreviations: ASAMI, association for the study and application of methods of ilizarov; E, excellent; F, fair; G, good; P, poor

The difficulty a lot of these patients experience is the longevity of referral and treatment. Potentially, they could be out of the labour market for at least two years which could impede their prospects of getting back to work. We echo other studies which recommend referral should be made to a limb reconstruction unit by six months. The patients that did not go back to work were in receipt of disability/incapacity benefits and did not feel working would offer them better remuneration. Our results for working are slightly poorer that our International counterparts; 82% Greece (10), 77% India (11), 86.7% Canada (12) but are better than some British studies; 22% 4. This could be attributed to the fact that other countries do not provide a generous Social Security System as we do so there is more incentive for them to work. Achieving union and eradicating infection at the same site can prove challenging. A diagnosis of non-union can be made when at least 6 months have elapsed with no evidence of progression of healing after the time of the fracture. At this point if there is an infection then it tends to be chronic and the organism tends to be resistant to most antibiotics (13, 14).

In order to eradicate the infection all of the necrotic bone needs to be completely resected. However, this can make bridging of the bones more difficult and moreover it is also associated with deformity, leg-length discrepancy, joint stiffness, disuse osteoporosis, neurovascular deficit and soft-tissue atrophy (10). In all our patients at the time of frame removal and subsequent follow up infection had been eliminated. Although the possibility of a flare up cannot be excluded the absence of recurrence of infection is a good result. In the current series our bone results were better than the functional results. This demonstrates that an excellent bone result does not necessarily guarantee good function. The functional result is affected by the soft tissues i.e conditions of the muscles, vessels and the joints. The effects of smoking on the outcome of ring fixation have already been reported (15). Half of our follow up patients were still smokers despite being warned of the risk of a poorer result. One person was a regular substance abuser at the time of treatment. However, they all still managed to complete the treatment successfully. Our numbers were too small to establish whether there was a difference in outcomes between smokers and non-smokers. All of the patients were able to walk and bear weight immediately after the application of the frame. Ilizarov considered this an essential principle in his treatment. One of our patients was 88 years of age when he had the Ilizarov frame. In mature patients there may be issues such as concomitant comorbidities which may affect treatment in the frame. Many older patients have osteoporotic or osteopaenic bone and the traditional external fixators would struggle to get adequate purchase (16-19). However, we did find that the tensioned wires and half pins did function well in this one patient. There are also issues of poor skin and soft tissue in older patients which could lead to suboptimal healing especially when prominent internal fixation is used. The ability to bear weight is a huge advantage for the older population where recumbancy can increase the risk of infections and thromboembolic disease. Amputation is an option for the older patient but it has been shown that they do not do as well as their younger counterparts (20-22). So the Ilizarov method provides a viable alternative to amputation.

The Lower Extremity Assessment Project (LEAP) (23) compared the outcomes following limb salvage versus amputation. In total eight level one trauma centres were included. They found there was no difference in the two year functional outcome after limb salvage and amputation. The financial implications were fairly similar at two years ($81316 salvage *vs *$91106 amputation). However, the lifetime cost was markedly different ($163 282 salvage *vs *$509275 amputation). We believe that limb reconstruction should be offered as a viable alternative to amputation (Table 6). 

**Table 6.** Lower Extremity Assessment Project Study Results

LEAP ^a^ study	Limb Salvage	Amputation
**2 years function**	None	None
**2 years financial cost, $**	81 316	91 106
**Lifetime cost, $**	163 282	509 275

^a^Abbreviation: LEAP, lower extremity assessment project

We also found that a multidisciplinary (MDT) approach was tantamount to our successful outcomes. Currently, our limb reconstruction service comprises a consultant specialist surgeon, consultant microbiologist, clinical nurse frames specialist and a dedicated physiotherapist. Patients had easy access to the clinical nurse specialist via a mobile phone if they had any concerns such as pin site infections. They were also education on their treatment throughout. This we feel partly accounts for the fact that all patients said they would have the procedure again.

### 5.1. Limitations of the Study

The main limitation to the study was our numbers. Initially twenty patients were identified but only twelve could be contacted and subsequently followed up. Our unit is relatively new and the total treatment time can take up to two years therefore we would not expect to have such high numbers.

Treatment of infected non unions of the tibia with the Ilizarov method is a viable alternative to amputation. Our results are comparable, if not better than most established units.
